# An Integrated Approach to Verify Spot Position and Isocenter in Image-Guided Proton Therapy

**DOI:** 10.1016/j.ijpt.2025.100755

**Published:** 2025-06-18

**Authors:** Riki Oshika, Shunsuke Moriya, Masashi Yamanaka, Kazuki Matsumoto, Takeji Sakae, Tomonori Isobe

**Affiliations:** a Degree Programs in Comprehensive Human Sciences, Graduate School of Comprehensive Human Sciences, University of Tsukuba, Ibaraki, Japan; b Proton Medical Research Center, University of Tsukuba, Ibaraki, Japan; c Department of Medical Physics, Shonan Kamakura General Hospital, Kanagawa, Japan; d Institute of Medicine, University of Tsukuba, Ibaraki, Japan

**Keywords:** Spot position verification, Cone-beam computed tomography, Image-guided proton therapy, X-ray CT-based polymer gel dosimetry, Spot scanning

## Abstract

**Purpose:**

To develop and validate a novel 3-dimensional quality assurance (QA) method using an X-ray computed tomography-based polymer gel dosimeter (XCT-PGD) and an integrated cone-beam computed tomography (CBCT) system, enabling comprehensive verification of spot positions and isocenter accuracy for proton therapy.

**Materials and Methods:**

A PROBEAT-M1 proton therapy system was used to irradiate an XCT-PGD with spot scanning beams from 4 gantry angles (45°, 180°, 270°, and 315°). Three spot positions, including the isocenter position, were evaluated. Pre- and postirradiation CBCT scans were acquired to determine the spot positions. The accuracy of the proposed method was validated against conventional Winston-Lutz tests and beam delivery log file analysis. Spot positions were analyzed using 3-dimensional visualization of the beam trajectories within the gel dosimeter.

**Results:**

The proposed method demonstrated excellent agreement with conventional techniques. The isocenter position determined using our method was within 0.39 mm that from the Winston-Lutz test. Spot position from our method was within 0.37 mm that from log file analysis. Three-dimensional detection using the gel dosimeter enabled simultaneous verification of multiple spot positions and beam trajectories, providing comprehensive spatial information not available through conventional 2-dimensional methods.

**Conclusion:**

Combining an XCT-PGD with CBCT provides a robust and comprehensive method for 3-dimensional verification of the accuracy of proton therapy delivery. This novel approach offers advantages over conventional QA techniques by enabling simultaneous evaluation of spot positions and isocenter accuracy while minimizing setup uncertainties. The method is particularly suitable for annual QA and commissioning procedures in proton therapy facilities.

## Introduction

Proton therapy is a radiation treatment modality that can deliver a high dose to the target while sparing normal tissues, owing to the unique physical characteristics of protons. The recent introduction of intensity-modulated proton therapy (IMPT) has enabled three-dimensional (3D) optimization of dose distribution in both distal and proximal regions, improving target conformity. However, these highly conformal dose distributions are susceptible to patient positioning errors and beam delivery inaccuracies.^1^ This challenge has been addressed by implementing routine image guidance using cone-beam computed tomography (CBCT) or X-ray fluoroscopy.^2^, ^3^ Nevertheless, reports^4^ have indicated that systematic spot position errors can significantly impact dose distributions in IMPT, making stringent quality assurance (QA) of the treatment system essential.

QA programs targeting beam parameters have been proposed.^5^ Accuracy of the spot position and isocentricity of the gantry and couch are critical QA items of beam parameters. Conventional QA methods use films, 2-dimensional array detectors, spot monitors or charge-coupled device cameras coupled with plastic scintillators or fluorescent screens to evaluate spot position accuracy and isocenter precision in mechanical coordinate systems defined by external lasers. However, these methods have several limitations: (1) QA items are evaluated individually, necessitating a comprehensive assessment of overall positioning accuracy; (2) evaluations are limited to 2 dimensions; and (3) direct evaluation in the image coordinate system is not performed, and even if surrogates are used, positioning uncertainties are included. To our knowledge, no research has directly and simultaneously evaluated isocenter and spot position accuracy in the image coordinate system.

X-ray CT-based polymer gel dosimeters (XCT-PGDs) have been developed and investigated for clinical applications in radiation therapy.^6^, ^7^, ^8^, ^9^ These gel dosimeters can be read using X-ray CT, eliminating the need for additional readout devices. Using a CBCT system integrated into the treatment system for readout, it is possible to perform dosimetry in the same image coordinate system for patient positioning, minimizing positional uncertainty.

This study proposes and evaluates the feasibility of a method using an XCT-PGD and a CBCT image guidance system routinely used in clinical practice for 3D verification of the accuracy of beam delivery to arbitrary spot positions in proton therapy. This novel approach aims to overcome the limitations of existing QA methods by providing a comprehensive, direct, and 3D evaluation of beam delivery accuracy in the image coordinate system.

## Materials and methods

### Measurement setup

**Figure 1:**
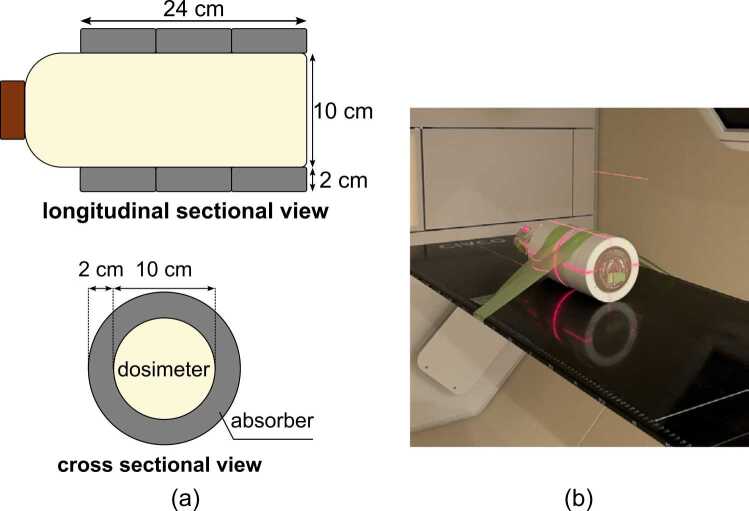
Experimental setup for the X-ray computed tomography-based polymer gel dosimeter (XCT-PGD). (a) Schematic diagram of the gel dosimeter in a cylindrical container placed inside an absorber. (b) Photograph of the actual setup on the treatment couch. The gel dosimeter is placed in an absorber and positioned using external lasers so that the center of its sensitive region aligns with the mechanical isocenter. The dosimeter is then secured in place with adhesive tape.

A commercially available XCT-PGD, dGEL (Triangle Products, Kashiwa, Japan), was used for measurements. These dosimeters are suitable for readout using on-board CBCTs in radiation therapy systems and have been used in previous studies to verify irradiation accuracy and dose distribution.^8^, ^9^ The experimental setup for the XCT-PGD is illustrated in Figure 1. The gel dosimeter was encased in a cylindrical container with a diameter of 10 cm and height of 25 cm. This container was then embedded in a 3D printed absorber made of polylactic acid with a thickness of 2.0 cm, as shown in Figure 1a. The absorber was specifically designed to prevent proton beam penetration to protect the laser alignment system positioned opposite to the beam port. In our specific proton therapy system configuration, without this absorber, transmitted proton beams could potentially damage the laser equipment used for patient setup. Notably, this absorber may not be necessary for other proton therapy systems. The dosimeter was positioned on the treatment couch, with the center of its sensitive volume aligned approximately to the mechanical isocenter, and secured using adhesive tape to prevent movement, as shown in Figure 1b. This approach differs fundamentally from conventional methods that rely on absolute positioning accuracy. Instead, it evaluates dose delivery within the image coordinate system using CBCT images, making precise alignment between the dosimeter and mechanical isocenter less critical for accurate results.

### Beam delivery and image acquisition

**Figure 2:**
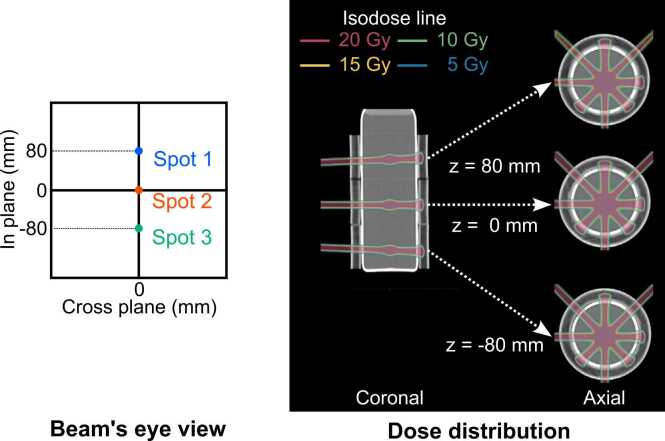
Treatment planning for X-ray computed tomography-based polymer gel dosimeter method. The treatment plan involves irradiating 3 spots at each of 4 gantry angles.

A PROBEAT-M1 proton therapy system (Hitachi High-Tech Corporation, Tokyo, Japan) was used to deliver proton beams and acquire CBCT images. Treatment planning was performed using the VQA Treatment Planning System (Hitachi High-Tech Corporation, Tokyo, Japan). Figure 2 illustrates the treatment plan for this study. Spots were set at 3 positions, including the isocenter. These spot positions were consistent across all gantry angles. Proton beams were delivered using spot scanning technique. Proton beams with an energy of 144.1 MeV and spot size of 3.48 mm (in-plane) and 3.63 mm (cross-plane) were delivered from the gantry angle of 180°, while proton beams with an energy of 139.0 MeV and spot size of 3.59 mm (in-plane) and 3.74 mm (cross-plane) were delivered from the other gantry angles (45°, 270°, and 315°). These energies were specifically selected to ensure that the proton beams would not penetrate through the gel dosimeter and its absorber. The spot sizes were measured in air on the isocenter plane. The proton energies were adjusted to ensure consistent water equivalent length to the isocenter plane across all gantry angles, accounting for the additional water equivalent length contributed by the treatment couch for the 180° beam. The monitor units for each proton beam were adjusted to deliver approximately 20 Gy of physical dose per beam. This high dose was chosen based on dGEL's limited dose-response characteristics (approximately 4 HU at 10 Gy and 8 HU at 20 Gy).^8^ While previous studies using the same XCT-PGD for treatment accuracy verification achieved satisfactory results with doses of 10 Gy,^9^ those studies utilized iterative CBCT algorithm, which offers superior low-contrast resolution compared to Feldkamp algorithm.^10^ We therefore used for a higher dose to ensure robust beam path visualization in our specific configuration.

CBCT imaging was performed twice, preirradiation and immediately .postirradiation. Both scans were acquired with identical parameters: tube voltage of 120 kV, tube current-exposure time product of 80 mAs, pixel resolution of 0.39 mm/pixel, and slice thickness of 2.0 mm. A total of 1223 projections were acquired for each image reconstruction. The Feldkamp algorithm was used for image reconstruction.

We used a 2.0 mm slice thickness, which is the standard protocol at our hospital. To assess method robustness, we also reconstructed images at 0.5 mm and 1.0 mm slice thicknesses and analyzed their impact on spot position detection (Supplementary Table A1). Maximum 3D displacement differences between slice thicknesses were within 0.3 mm, well below the 1 mm tolerance specified by AAPM Task Group 224 guidelines.^5^ Variations in slice thickness had minimal clinical impact on verification results. Based on these findings, we adopted the 2.0 mm slice thickness for comparison with other QA methods.

### Analysis

Data were analyzed with an in-house program developed using Python 3.11, with optimization procedures implemented via the SciPy module. We applied the analysis methods of previous studies on the verification of linear accelerator treatment isocenters using CBCT images of photon-irradiated gel dosimeters,^9^, ^11^ with modifications. In these methods, the contrast-to-noise ratio obtained from the subtraction images of the pre- and postirradiation CBCT scans is used to optimize and detect the 3D beam path. A key modification was the application of a 3D cross-directional bilateral filter to both pre- and postirradiation CBCT images during preprocessing. This filter effectively reduces noise while preserving edge information,^12^ enhancing the accuracy of beam path detection. We also modified the analysis to account for energy-dependent spot sizes when creating regions of interests for analysis, ensuring that beam size variations across different proton energies were properly incorporated into the detection algorithm. Another significant adaptation was measuring the spot position ($p_{\mathrm{measure}}$) relative to the imaging coordinate system, rather than calculating the isocenter. This was achieved by minimizing the following objective function on the basis of the detected beam axes:

$$\underset{p_{\mathrm{measure}}}{\arg\min}\left(\underset{i}{\max}d_{l_{i}}\left(p_{\mathrm{measure}}\right)\right),$$

where $d_{l_{i}}(p)$ represents the distance from point p to the detected beam axis $l_{i}$.

### Comparison with conventional methods

To validate our proposed method with a comparative analysis, QA of the beam parameters was also performed using 2 conventional QA techniques: spot position measurement using log files and isocenter testing using a metal ball and film.

The spot position was tested using log files from the spot monitor mounted on the gantry. Proton beams with an energy of 144.1 MeV and spot size of 3.48 and 3.63 mm in the in-plane and cross-plane directions, respectively, were irradiated from 4 gantry angles, identical to those used in XCT-PGD method. The spot monitor measured the spot positions corresponding to the positions on the monitor plane, which were recorded in a log file. To transform these values from the monitor plane to the isocenter plane, a similarity ratio was applied according to the geometric projection relationship between the 2 planes. This coordinate transformation is represented by the following equation:

$$p_{\mathrm{isocenter}}=p_{\mathrm{monitor}}\;\times \frac{\mathrm{SAD}}{\mathrm{SMD}}$$

where $p_{\mathrm{isocenter}}$ and $p_{\mathrm{monitor}}$ are the position coordinates on the isocenter plane and monitor plane, respectively; SAD is the distance from the radiation source to the isocenter plane (cross-plane: 135 cm, in-plane: 192.5 cm in the PROBEAT-M1 system); and SMD is the distance from the radiation source to the monitor plane (cross-plane; 78 cm, in-plane; 192.5 cm). The accuracy of the spot position monitors has been validated during annual QA procedures, where spot positions recorded in the log files are compared with those measured on film during Winston-Lutz test.^13^ These comparisons show differences within 0.2 mm at the isocenter position.

The conventional isocenter test, also known as the Winston-Lutz test,^13^ was performed using a metal ball and film for a direct comparison, particularly for Spot 2 (isocenter). In this procedure, a metal ball was first aligned with the imaging isocenter using orthogonal X-ray imaging. Subsequently, proton beams with an energy of 144.1 MeV and spot size of 3.48 mm (in-plane) and 3.63 mm (cross-plane) were irradiated from 4 gantry angles, identical to those used in our method. For each gantry angle, the displacement between the center of the metal ball's shadow and the center of the spot on the film was measured. These displacements were then analyzed to determine the treatment isocenter position.

## Results

Subtracting the pre- and postirradiation CBCT images revealed that irradiated volumes of the XCT-PGD corresponding to the beam paths increased in CT value, as shown in Supplementary Figure A1. Thus, the gel dosimeter provided clear visualization of the irradiation patterns.

Table 2 summarizes the detected spot positions using XCT-PGD method compared with the planned positions. The displacement was within 1 mm for all spot positions, with a maximum displacement of 0.62 mm. Larger displacements were observed at off-isocenter positions (spots 1 and 3) compared to the isocenter position (spot 2). The magnitude of displacement was 0.35 mm at the isocenter position, while the off-isocenter positions showed displacements of 0.64 mm and 0.56 mm for spots 1 and 3, respectively. The detected spot had a maximum radius of 0.30 mm, indicating excellent field isocentricity.

**Table 1:** Cone-beam computed tomography (CBCT) image acquisition and reconstruction parameters.

Acquisition parameters		Reconstruction parameters	
X-ray voltage	120 kV	Base method	Feldkamp
Tube current-exposure time product	80 mAs	Resolution	0.39 mm/pixel
Number of projections	1223	Slice thickness	2.0 mm

**Table 2:** Comparison of detected using XCT-PGD method and planned spot positions.

	Planned spot position (mm)			Difference from planned to detected spot position (mm)				Detected spot radius (mm)
	x (L-R)	y (P-A)	z (S-I)	x (L-R)	y (P-A)	z (S-I)	Magnitude	
Spot 1	0.00	0.00	80.00	0.34	0.51	0.11	0.62	0.30
Spot 2	0.00	0.00	0.00	0.05	0.33	0.11	0.35	0.17
Spot 3	0.00	0.00	−80.00	0.17	0.46	−0.28	0.56	0.20

**Abbreviations:** XCT-PGD, X-ray CT-based polymer gel dosimeter; L, left; R, right; P, posterior; A, anterior; S, superior; I, inferior.

**Figure 3:**
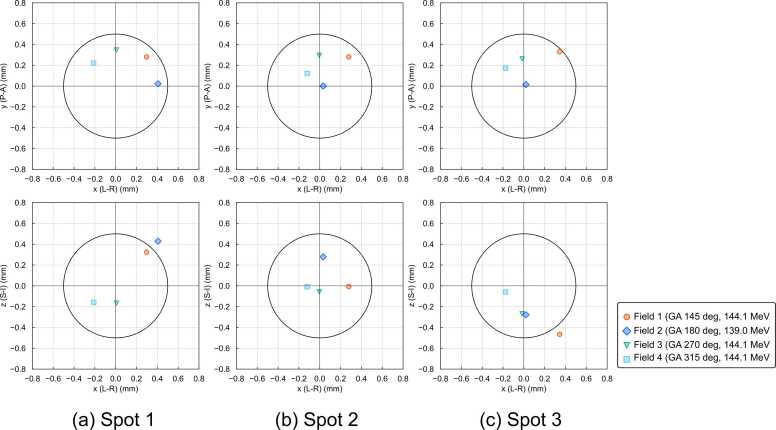
Three-dimensional displacement of field positions from planned spot positions measured using the XCT-PGD method. The black circle in each subplot represents a 0.5 mm radius. Abbreviations: L, left; R, right; P, posterior; A, anterior; S, superior; I, inferior; GA, gantry angle.

Figure 3 shows the 3D displacement of field positions from the planned spot positions measured using XCT-PGD method. All field displacements were within 1 mm, with a maximum displacement of 0.67 mm.

Table 3 compares the results from XCT-PGD method with those from log file QA. The results from XCT-PGD method were calculated from the intersection of the 3D detected field in the imaging coordinate system and the plane passing through the isocenter and perpendicular to the beam axis. In contrast, log file QA results were derived from the spot monitor mounted on the gantry. The results from XCT-PGD method and log file QA agreed within 0.37 mm.

**Table 3:** Comparison of field position detected using XCT-PGD method and the log file method.

	Planned spot position		Gantry angle (degree)	XCT-PGD method		Log file		Difference	
	Cross-plane (mm)	In-plane (mm)		Cross-plane (mm)	In-plane (mm)	Cross-plane (mm)	In-plane (mm)	Cross-plane (mm)	In-plane (mm)
Spot 1	0	80	45	0.41	0.32	0.17	0.04	0.24	0.28
			180	−0.41	0.43	−0.04	0.17	0.37	0.26
			270	−0.35	−0.17	−0.03	0.29	0.31	−0.13
			315	−0.31	−0.16	0.05	0.13	0.25	0.03
Spot 2	0	0	45	0.40	−0.01	0.16	0.14	0.24	−0.13
			180	−0.03	0.28	−0.10	0.27	−0.07	0.01
			270	−0.30	−0.06	−0.09	0.38	0.21	−0.33
			315	−0.17	−0.01	0.01	0.20	0.16	−0.20
Spot 3	0	−80	45	0.48	−0.47	0.12	0.24	0.35	0.22
			180	−0.02	−0.28	−0.11	0.39	−0.09	−0.11
			270	−0.26	−0.27	−0.13	0.48	0.13	−0.21
			315	−0.25	−0.06	−0.04	0.30	0.20	−0.24

Note: XCT-PGD method directly evaluates field positions in the imaging isocenter coordinate system, whereas the log file method uses positions detected by the spot monitor mounted on the gantry.

**Abbreviation:** XCT-PGD, X-ray CT-based polymer gel dosimeter.

Table 4 shows the results for Spot 2 (isocenter) from XCT-PGD method and the Winston-Lutz test using a metal sphere and film. These results agreed within 0.39 mm.

**Table 4:** Comparison of isocenter positions detected using XCT-PGD method and the Winston-Lutz test.

XCT-PGD method (mm)			Winston-Lutz test (mm)			Difference (mm)		
x (L-R)	y (P-A)	z (S-I)	x (L-R)	y (P-A)	z (S-I)	x (L-R)	y (P-A)	z (S-I)
0.05	0.33	0.11	0.08	−0.06	−0.22	−0.03	0.39	0.33

Note: XCT-PGD method directly evaluates the imaging isocenter, whereas the Winston-Lutz test relies on aligning a metal ball with the imaging isocenter.

Differences between planned and detected spot positions at the isocenter (Spot 2) are shown.

**Abbreviations:** XCT-PGD, X-ray CT-based polymer gel dosimeter; L, left; R, right; P, posterior; A, anterior; S, superior; I, inferior.

## Discussion

This study presents a novel method using an XCT-PGD with on-board CBCT for comprehensive 3D verification of proton beam delivery accuracy. Our approach offers several advantages over conventional QA techniques, by allowing spot position verification within the CBCT coordinate system routinely used for image guidance. This integration enables a streamlined verification process with minimal alignment uncertainty, while providing intuitive 3-dimensional evaluation of results.

Our method demonstrated excellent agreement with conventional techniques—the isocenter position was within 0.39 mm of the Winston-Lutz test results, and spot positions were within 0.37 mm of log file QA measurements. A fundamental difference between these approaches is dimensional: our method detects complete 3D beam trajectories, whereas conventional tests only identify beam intersection points with planes perpendicular to the gantry angle in 2 dimensions. This enables our method to detect oblique beam incidence caused by gantry misalignment or mechanical sag that 2D methods might miss when beams appear accurate on the measurement plane despite angular errors. The larger discrepancies observed at off-isocenter positions compared to the isocenter position (0.64/0.56 mm vs 0.35 mm) further support this advantage, as conventional QA methods using gantry-mounted spot monitors cannot independently verify gantry rotation accuracy in an absolute coordinate system.

The agreement between our method and conventional approaches is well below the 1 mm tolerance specified by AAPM Task Group 224 guidelines^5^ for spot and isocenter position accuracy. Combined with the 0.4 mm reproducibility reported in previous XCT-PGD studies for linear accelerators,^9^ this validates our approach for clinical implementation using established tolerance values. Accurate spot position verification is particularly crucial for IMPT, as demonstrated by Yasui et al,^14^ who showed that systematic spot position errors of ±2 mm in head and neck IMPT can cause brainstem maximum dose variations of up to 6.20% even with worst-case optimization. Our method's ability to evaluate multiple off-center positions simultaneously provides valuable verification for treatments targeting regions away from the isocenter.

Practical implementation requires consideration of certain factors. Due to XCT-PGDs' relatively small CT value increases with dose, high radiation doses (20 Gy isodose line) are needed to clearly visualize the beam paths, making this requirement, we suggest that this method is more suitable for annual QA or commissioning processes rather than frequent evaluations to balance thoroughness with practicality.

Though our study focused on specific energies, the method's applicability to different proton energies is possible through our software's ability to adjust analysis parameters based on varying spot sizes. The approach can be extended to evaluate noncoplanar irradiation accuracy by incorporating couch rotation. A limitation of our current approach is that dosimeter shifts might occur during couch movement. To address this limitation and facilitate broader adoption, we propose future improvements including modifying the absorber with a flat bottom surface and adding mounting features that securely attach to the treatment couch, thereby enhancing both convenience and reliability of this method. These enhancements would allow for a more comprehensive assessment of isocenter accuracy in a single session, further extending the utility of XCT-PGD method.

Our results were obtained using a specific type of gel dosimeter (dGEL) and a single CBCT system and proton therapy system (PROBEAT-M1), which limits cross-system validation. However, since proton interactions follow consistent physical principles, we believe this approach could be applied to any spot scanning proton system with CBCT or in-room CT capabilities, though implementation at other combinations would require initial commissioning to establish system-specific parameters. Further research is needed to validate the method across different dosimeter formulations and dosimeters, imaging systems, and proton therapy platforms is needed to ensure its confirm broad applicability and reliability across various proton therapy setups.

## Conclusion

This study presents a novel, comprehensive 3D method using an XCT-PGD and CBCT for spot scanning proton therapy system QA, addressing key limitations in current techniques for beam delivery verification. By offering a more comprehensive and intuitive assessment of system accuracy in the imaging coordinate system, our approach has the potential to enhance the precision and safety of spot scanning proton therapy.

## Author Contributions

Riki Oshika: Methodology, Software, Writing- Original draft. Shunsuke Moriya: Validation, Writing- Review and Editing. Masashi Yamanaka: Methodology, Writing- Review and Editing. Kazuki Matsumoto: Methodology, Writing- Review and Editing. Takeji Sakae: Project administration, Writing- Review and Editing. Tomonori Isobe: Supervision, Writing- Review and Editing.

## Declaration of Conflicts of Interest

The corresponding author Shunsuke Moriya serves as a technical advisor for Triangle Products Co, Ltd and has received an honorarium from the company. The other authors have no conflicts of interest to disclose. The other authors have no conflicts of interest to disclose. Despite these relationships, the authors affirm that the work presented in this publication was conducted with integrity and without undue influence from the above entities.

## Declaration of Generative AI and AI-assisted technologies in the writing process

During the preparation of this work, the author used DeepL to improve language and readability only. After using this tool/service, the author reviewed and edited the content as necessary and is fully responsible for the content of the publication.

## Data Availability Statement

Research data are stored in an institutional repository and will be shared upon request to the corresponding author.

## Footnotes

Appendix A Supplementary data associated with this article can be found in the online version at doi:10.1016/j.ijpt.2025.100755.
