# Supplementary material for: An Integrated Approach to Verify Spot Position and Isocenter in Image-Guided Proton Therapy
Source: Int J Part Ther. 2025 Jun 18;17:100755. doi: 10.1016/j.ijpt.2025.100755 (PMC12270928; doi:10.1016/j.ijpt.2025.100755)
Supplement: Supplementary file 1 — Supplementary material [file mmc1.pdf]

An Integrated Approach to Verify Spot Position and Isocenter in Image-Guided Proton Therapy

Riki Oshika, MS[1,2], Shunsuke Moriya, PhD[3], Masashi Yamanaka, PhD[4], Kazuki Matsumoto, MS[1,4], Takeji Sakae, PhD[3], Tomonori Isobe, PhD[5]

1. Degree Programs in Comprehensive Human Sciences, Graduate School of Comprehensive Human Sciences, University of Tsukuba, Ibaraki, Japan
2. Radiation Safety and Quality Assurance Division, National Cancer Center Hospital, Tokyo, Japan
3. Proton Medical Research Center, University of Tsukuba, Ibaraki, Japan.
4. Department of Medical Physics, Shonan Kamakura General Hospital, Kanagawa, Japan
5. Faculty of Medicine, University of Tsukuba, Ibaraki, Japan.

Corresponding Author

Shunsuke Moriya PhD

Proton Medical Research Center, University of Tsukuba, Ibaraki 305-8576, Japan.

E-mail: smoriya@md.tsukuba.ac.jp

Supplementary Material

Supplementary Table A1 Comparison of differences between planned and detected spot positions using the XCT-PGD method with CBCT images reconstructed at different slice thicknesses.

	Spot 1				Spot 2				Spot 3			
slice thicknesses	x (L-R) (mm)	y (P-A) (mm)	z (S-I) (mm)	Magnitude (mm)	x (L-R) (mm)	y (P-A) (mm)	z (S-I) (mm)	Magnitude (mm)	x (L-R) (mm)	y (P-A) (mm)	z (S-I) (mm)	Magnitude (mm)
0.5 mm	0.17	0.36	0.68	0.79	0.12	0.30	0.05	0.32	0.19	0.35	0.31	0.51
1.0 mm	0.20	0.39	0.36	0.57	0.23	0.27	-0.16	0.39	-0.01	0.29	-0.10	0.30
2.0 mm	0.34	0.51	0.11	0.62	0.05	0.33	0.11	0.35	0.17	0.46	-0.28	0.56

Abbreviations: XCT-PGD, X-ray CT-based polymer gel dosimeter; L, left; R, right; P, posterior; A, anterior; S, superior; I, inferior.

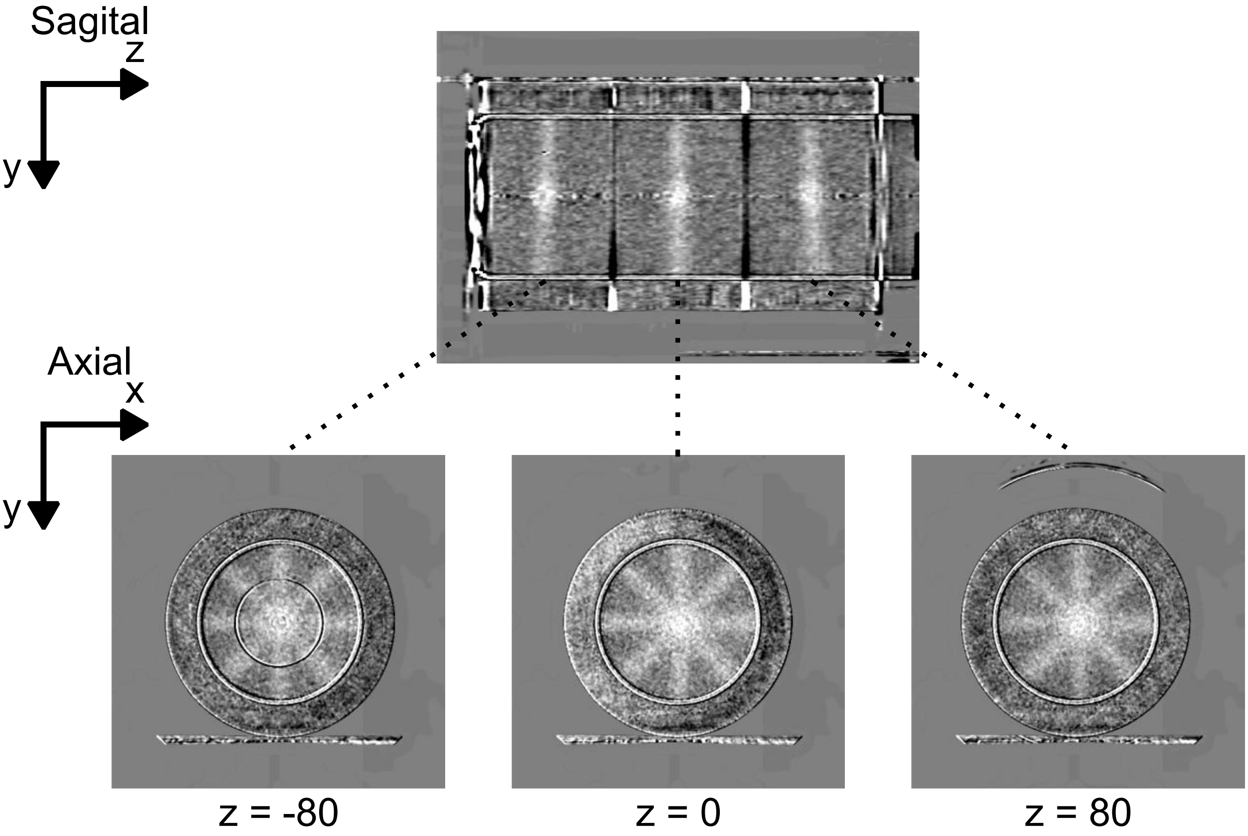

Supplementary Figure A1 Subtraction image of pre- and post-irradiation CBCT images of a gel dosimeter.

The window level and width were set to 0 and 40, respectively.
